# Host Status of Persian Lime (*Citrus latifolia* Tan.) to Oriental Fruit Fly and Mediterranean Fruit Fly (Diptera: Tephritidae) in Hawai’i

**DOI:** 10.3390/insects15100799

**Published:** 2024-10-14

**Authors:** Peter A. Follett, Xiuxiu Sun, Spencer S. Walse

**Affiliations:** 1 USDA-ARS, Daniel K. Inouye U.S. Pacific Basin Agricultural Research Center, 64 Nowelo St., Hilo, HI 96720, USA; xiuxiu.sun@usda.gov; 2 USDA-ARS, San Joachim Valley Agricultural Research Center, 9611 S. Riverbend Ave., Parlier, CA 93648, USA

**Keywords:** citrus, nonhost, host status, quarantine, phytosanitary, *Bactrocera dorsalis*, *Ceratitis capitata*

## Abstract

**Simple Summary:**

Tephritid fruit flies are major economic pests that impact fruit production and impede international trade. The type of host fruit influences the ability of a fruit fly to complete their life cycle. International regulatory standards that define host status categorize fruits as a natural host, a conditional host, or a nonhost. For those fruits that are natural or conditional hosts, the infestation rate can vary across a spectrum ranging from highly attractive well-suited hosts supporting large numbers of fruit flies to very poor hosts supporting low numbers. Persian lime, *Citrus x latifolia*, is a new crop in Hawai’i, and no information existed on its susceptibility to Hawai’i’s tephritid fruit fly pests. Host status testing was conducted using no-choice laboratory and field cage tests as well as field collection of fruit. Mediterranean fruit fly and Oriental fruit fly oviposited and developed in artificially damaged (punctured) limes in cage tests but did not infest undamaged commercial quality fruit, suggesting Persian limes should be considered a conditional host. Field-collected and processed export-quality Persian limes (total of 45,954 fruit) were not naturally infested by Mediterranean fruit fly and Oriental fruit fly. In regulations, risk managers may use the term conditional nonhost to describe regulated articles, like Persian limes from Hawai’i, which do not pose a pest risk.

**Abstract:**

We investigated the host status of harvest-ready green Persian lime, *Citrus x latifolia* Tan. (Rutaceae), to Oriental fruit fly (*Bactrocera dorsalis* [Hendel]) and Mediterranean fruit fly (*Ceratitis capitata* [Wiedemann]) (Diptera: Tephritidae) using laboratory and field studies. In forced-infestation small cage exposures (using 25 × 25 × 25 cm screened cages with 50 gravid females) and large olfactometer cage tests (using 2.9 × 2.9 × 2.5 m walk-in screened cages with 100 gravid females), punctured limes were infested by Oriental fruit fly and Mediterranean fruit fly at low rates compared to papaya controls, whereas undamaged intact fruit was not infested. Field collection and packing of 45,958 commercial export-grade fruit and subsequent incubation to look for natural infestation resulted in no emergence of fruit flies. Forced infestation studies in the field using sleeve cages to enclose fruit with a high density of fruit flies (50 gravid females) on the tree also showed no infestation. Commercial export-grade Persian lime fruit should be considered a conditional nonhost for Oriental fruit fly and Mediterranean fruit fly.

## 1. Introduction

Limes are a nutrient-rich and popular citrus fruit used primarily to accent foods and beverages. Persian lime (*Citrus x latifolia* Tan., also called Tahiti lime) is the most widely cultivated lime species commercially. Persian lime is a seedless hybrid between Key lime [*Citrus x aurantiifolia* [(Christm.) Swingle] and lemon [*Citrus x limon* [(L.) Osbeck] [1]. Trees are adapted to a subtropical or tropical climate and produce fruit that are 3 to 6 cm in diameter with thin peels and light green, tender, acidic pulp. Mexico is the world’s largest producer of Persian limes, with an estimated production of 2.5 million tons, and the primary supplier to the United States, accounting for 92.6% of imports in 2022 [2].

Persian lime is a new crop in Hawai’i, with approximately 4000 ha recently planted on the island of Maui by Mahi Pono (Kahului, HI, USA). Fruit can be harvested year round, and once in full production, harvest volumes will exceed local demand and, therefore, export options are desirable. Citrus including Persian limes cannot be exported from Hawai’i to the continental United States without a quarantine treatment or other mitigation measures to control possible infestation by several federally regulated fruit flies, including Mediterranean fruit fly, *Ceratitis capitata* (Wiedemann), Oriental fruit fly, *Bactrocera dorsalis* (Hendel), and melon fly, *Zeugodacus cucurbitae* (Coquillet) (Diptera: Tephritidae). Different species and varieties of *Citrus* can vary widely in their suitability as hosts for fruit flies [3,4]. Persian lime is listed as a host for Oriental fruit fly and Mediterranean fruit fly but not for melon fly [5,6,7]. Two standalone quarantine treatments—irradiation and vapor heat—are approved to control fruit flies in several *Citrus* spp. (including *C. paradisi* Macfad. [grapefruit], *C. limon* [lemon], *C. aurantiifolia* [Key lime], *C. sinensis* (L.) Osbeck [orange], *C. grandis* (Burm.) Merr. [pummelo], and *C. noblis* var. *deliciosa* (Ten.) Swingle [tangerine]) for export from Hawai’i to the continental United States [6,8], but *C. latifolia* [Persian lime] was not included in the initial pest risk analysis for Hawai’i *Citrus* and so requires further study. 

An important part of pest risk analysis is determining the host status of a species or variety of fruit for regulated high-risk pests associated with the fruit, especially fruit flies, and such determinations are required before developing export protocols for a new fruit in international or domestic trade [9]. Hosts for fruit flies are fruits on which flies can lay eggs and complete their whole life cycle through to the emergence of adults of the next generation [10,11]. Any plants that do not allow flies to produce viable adult offspring are, therefore, by definition, a nonhost. Fruits that are nonhosts pose no threat of supporting an invasion by a fruit fly, and those fruits can be safely traded without phytosanitary measures. 

Determining the host status of a fruit to a particular fruit fly experimentally often begins with a forced infestation cage exposure to determine the potential for infestation [12,13]. If adults are produced from the target fruit in forced cage exposures, further testing in the field, such as field cage exposures and collection of fruit from the tree, is recommended to determine if fruits are naturally infested [3,9]. Host testing for Persian limes has been conducted with several economically important species of tephritid fruit flies. Persian limes collected from commercial farms in Mexico showed no natural infestation by Mexican fruit fly, *Anastrepha ludens* Loew [14]. Persian limes placed in an oviposition cage with Caribbean fruit fly, *Anastrepha suspensa* (Loew) did not become infested [15]. No Caribbean fruit flies were detected in a total of 102,384 field-collected Persian limes from 184 different groves in Florida; fruit samples included damaged, deformed, and rotted fruits in addition to sound yellow and green fruit [16]. Field-collected Persian limes in Brazil were not infested by South American fruit fly, *Anastrepha fraterculus* Wiedemann, or Mediterranean fruit fly [17]. Conversely, the Pacific fruit fly, *Bactrocera xanthodes* (Broun), and *B. kirki* (Frogg.) were reared from Persian (Tahitian) lime in laboratory cage tests with purposely damaged fruit in Western Samoa [18]. These results suggest that Persian limes may be generally a poor host or nonhost for tephritid fruit flies but can be suitable for fruit fly infestation under certain conditions. No information is available on the host status of Persian limes to Oriental fruit fly or Mediterranean fruit fly.

Systems approaches are increasingly being used to provide access to restricted markets in domestic and international trade. Systems approaches integrate two or more independent phytosanitary measures to cumulatively control pests in the pathway of the regulated article and provide quarantine security. Many systems-approach programs that have been developed include poor host status of the fruit [3,19]. In a retrospective analysis of 60 such protocols, poor host status was a component in 73% of all systems approaches targeting fruit flies [20]. A systems approach for exporting Persian lime from Hawai’i will be strengthened if a poor host or nonhost status can be confirmed for fruit flies.

Persian limes are picked and marketed while the peel is still green. At room temperature, the fruit can become yellow within a few days, which reduces its commercial marketability. With cold storage at 10 °C, Persian limes may have a 6–8-week shelf life [1,8]. In the present study, we evaluated the host status of commercial quality Persian limes to Oriental fruit fly and Mediterranean fruit fly using no-choice cage experiments and field collections. The goal was to determine whether Persian lime could be exported with minimal risk using a nonhost status protocol or using a systems approach built around its poor host status.

## 2. Materials and Methods

### 2.1. Experimental Insects and Fruit

Oriental fruit flies and Mediterranean fruit flies used in small and large cage exposure tests were obtained from colonies maintained at the USDA-ARS, United States Daniel K. Inouye Pacific Basin Agricultural Research Center in Hilo, Hawai’i on standard diets [21]. Fruit flies used in our tests were maintained in an insectary at 24–27 °C, 65–70% RH, and a photoperiod of 12:12 (L:D) hours. These fruit fly colonies have been maintained in large numbers (~50,000 adults per generation) for 20–30 years (~200–400 generations). For each test, approximately 1500 flies were transferred from large oviposition cages to smaller containers and moved to a cold room to allow separation of male and female flies. Female fruit flies were held in 40 mL plastic containers for up to three hours before testing and supplied with a 3:1 mixture of sucrose and enzymatic yeast hydrolysate (United States Biochemical, Cleveland, OH, USA) as a food source and given water ad libitum. Adult flies were 12 to 14 days old at the time of testing to ensure that females would be reproductively mature and actively laying eggs. All experiments were conducted with both Oriental fruit fly and Mediterranean fruit fly.

All Persian limes used in the experiments were grown at Mahi Pono farm, Kahului, HI (island of Maui) (elevation, 20 m; 20.860449° N, −159.4420898° W). Depending on the experiment, fruits were either harvested and shipped overnight to the USDA-ARS laboratory in Hilo, Hawai’i (island of Hawai’i) (for small and large semi-natural cage tests) or evaluated on the farm (in sleeve cage tests and large-scale field collections). Individual fruits weighed 90–120 g and were green or mostly green with a patch of yellow and uniform in shape. Locally grown papayas (*Carica papaya* L.) cv. ‘Rainbow’ (Pam Lee Trading Co., Hilo, HI, USA) that were 3/4 to fully ripe were used as positive controls and weighed 390 to 517 g. Host testing experiments and data analysis combined the approaches proposed in Cowley et al. (1992) [12], International Standards for Phytosanitary Measures (ISPM) No. 37 (FAO 2006) [9], and Follett and Hennessey (2007) [13].

### 2.2. Forced Infestation in Small Cages

**Figure 1:**
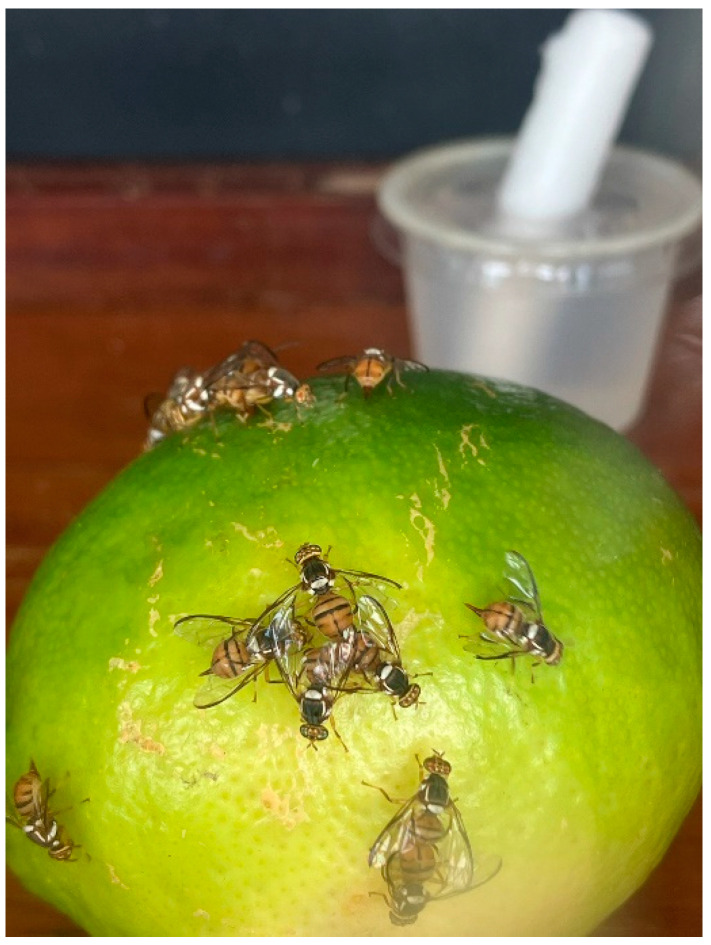
Small cage-forced infestation test with Oriental fruit flies attempting to oviposit in Persian lime.

Fruits were exposed to fruit flies at the USDA-ARS, U.S. Pacific Basin Agricultural Research Center in Hilo, Hawai’i. This laboratory is located on the windward side of the Big Island of Hawai’i at an elevation of 100 m. Trials were conducted from February to April 2024. All experiments were conducted outdoors under semi-natural conditions using screened cages placed on wooden shelves under a roof with sky lighting. Natural light was 11:13 (L:D) h, and temperature varied between 16.5 and 25 °C. Individual Persian lime fruits were placed on the floor of a 25 × 25 × 25 cm screen cage with 50 gravid female fruit flies for 24 h in a no-choice test following Cowley et al. (1992) [8] (Figure 1). All fruits were commercial grade U.S. No. 1 and No. 2 [22] and initially without damage or blemishes on the peel (i.e., undamaged). Fruits were washed before use in experiments. Fruits in separate tests were either punctured to simulate damage or left unpunctured. Punctures were made with a 1.0 mm diam. probe to a depth of 1 cm at 10 locations around the equator of the fruit. Unpunctured fruit had the peel intact and undamaged. The 24 h exposure period was used to maximize the chance of oviposition and infestation. On each test date, eight to twelve cages were set up each with an individual lime, and two cages were set up with a papaya fruit as a preferred host (positive control) to demonstrate oviposition competence [3]. This test was replicated on four dates with undamaged fruit and four dates with damaged fruit for a total of 36–48 test fruits for each damage treatment for each fruit fly species. After 24 h of exposure to either Oriental fruit fly or Mediterranean fruit fly, the fruit from each cage was placed in a 3.8 L plastic bucket with a screened lid and held at 20–25 °C. Approximately 50 g of sand was added to the bucket as a pupation medium. At two weeks and three weeks post-infestation, the sand was sieved and inspected for pupae, and pupae were transferred to 120 mL plastic cups for adult emergence. After three weeks, Persian lime and papaya fruits were dissected and examined for any remaining larvae.

### 2.3. Forced Infestation in Large Olfactometer Cages

**Figure 2:**
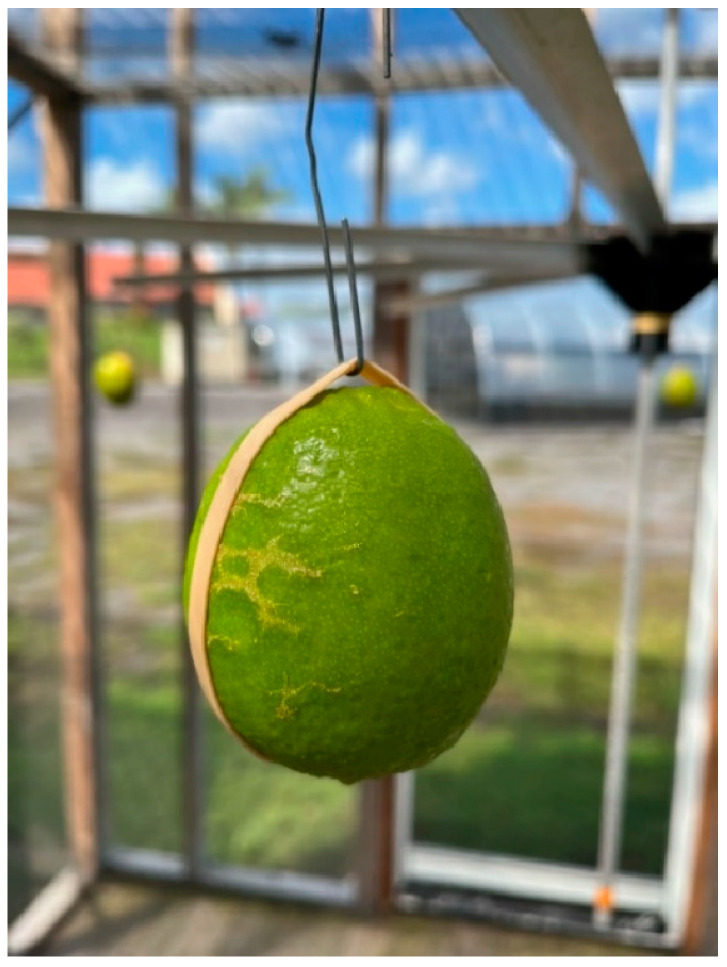
Large olfactometer cage with suspended Persian lime fruit.

In a set of four 2.9 × 2.9 × 2.5 m walk-in olfactometer screen cages, a single carousel was centered 1.8 m above the floor of each cage to rotate (~1 revolution min^−1^) four equally spaced fruits (Figure 2). Three replicate cages were used on each test date, and a fourth cage held a single papaya on the carousel as a preferred host (positive control). The experiment was replicated 12 times with undamaged fruit and 12 times with damaged fruit for a total of 48 fruit each. After 24 h of exposure to either 100 gravid Oriental fruit fly or 100 gravid Mediterranean fruit fly females, the four limes or one papaya from each cage were placed in 3.8 L plastic buckets with a screened lid and held at 20–25 °C for adult emergence as described above.

### 2.4. Forced Infestation in Sleeve Cages on the Tree 

**Figure 3:**
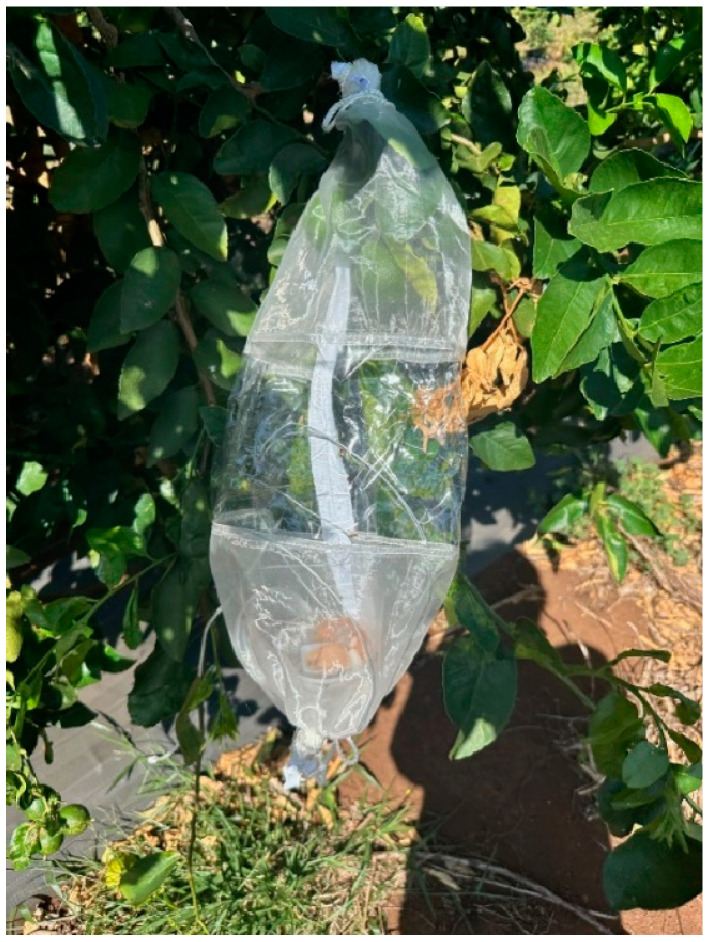
Sleeve cage over Persian limes on the tree with fruit flies inside the cage.

Lime fruits on trees were exposed to Oriental fruit fly and Mediterranean fruit fly using sleeve cages following the guidelines in ISPM 37 [10] (FAO 2006) in April–May 2024. In the field, groups of 3–4 harvest-ready green fruit on trees at Mahi Pono were enclosed with cylindrical (45 × 20 cm, height × diam.) fine mesh sleeve cages (BugDorm, Mega View Science, Taichung, Taiwan) (Figure 3). Ten different trees were selected for the Oriental fruit fly test and another ten trees were selected for the Mediterranean fruit fly test, with each tree receiving only one sleeve cage. Trees had not received any foliar sprays with pesticides for more than a month. Fifty gravid female flies of a given species were added to each sleeve cage for 24 h under ambient field conditions (temperature low and high values were 18–27 °C) as in the laboratory cage tests. As a control to demonstrate fly ovipositional competency, three additional trees were selected for each fruit fly species, and branches with fruits were enclosed with sleeve cages, but the fruits were intentionally damaged by making a 4–5 cm gash with a knife before adding 50 gravid females. After 24 h of exposure to Oriental fruit flies or Mediterranean fruit flies, undamaged and damaged fruits were harvested from trees, placed in sealed bins, and taken back to the laboratory at USDA ARS in Hilo, HI. In the laboratory, fruits were transferred into plastic containers with sand and held for fruit fly development as described above. Trees were approximately 2 m tall, and harvest-ready fruits were selected at 1.0 to 1.5 m height from the ground, choosing fruit mostly based on quality and ease of access for fitting sleeve cages.

### 2.5. Field Collection and Incubation of Fruit 

#### 2.5.1. Small-Scale Test

Lime fruits were sampled from trees at Mahi Pono Farm during harvest, shipped to the USDA-ARS laboratory in Hilo, HI, and observed for natural infestation following the guidelines in ISPM 37 [9]. Limes from the tree and limes from off the ground beneath the tree were collected randomly from multiple trees on five dates between January and March 2024 and were shipped to the USDA-ARS laboratory in Hilo the next day. Trees had not received any foliar sprays with pesticides for more than a month. Fruits from the tree were harvest ready with no obvious damage, and fruits off the ground had recently fallen (<two weeks on the ground) and were overripe with breaks in the skin but no obvious decay. Fruits were held in groups of 60–100 in unventilated fiberglass containers in the laboratory for fruit fly emergence as described above. McPhail traps baited with protein bait lures (torula yeast in water) were suspended at 64 locations in 32 orchard blocks at Mahi Pono farm to monitor fruit fly populations and demonstrate their presence on the farms during the time of fruit sampling. Trap captures were recorded biweekly. Fruit samples were collected from four orchard blocks with the highest trap captures of fruit flies.

#### 2.5.2. Large-Scale Test

**Figure 4:**
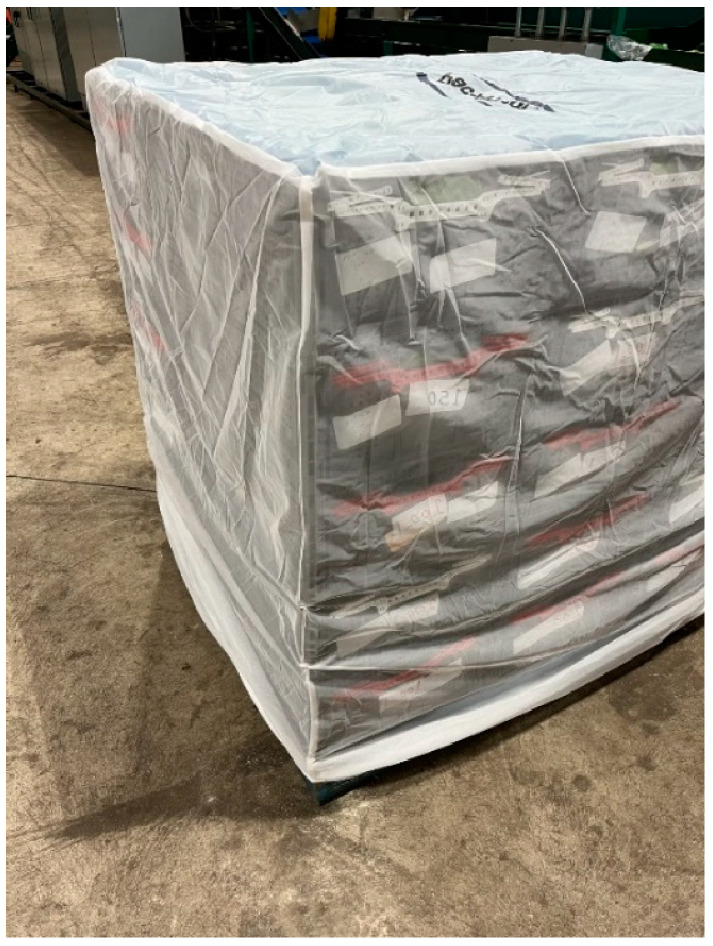
Pallet of field-collected Persian limes with a protective shroud.

A large-scale test was conducted in March 2024 by collecting approximately 10,000 fruits from each of the five orchard blocks and holding the fruit for the emergence of any fruit fly species. Fruits were harvested from trees that had not received any foliar sprays with pesticides for more than a month. The collected fruits from different blocks were combined and subjected to the Mahi Pono commercial packing line where the fruit received a fungicide spray, waxing, sorting into standard No. 1 (25%) and No. 2 quality (75%) fruit, and sizing based on the number of fruits that fit in a standard 30 lb [13.6 kg] box. Fruits were transferred into fifty 30 lb (13.6 kg) totes, stacked, palletized, shrouded to prevent the escape of any fruit flies or reinfestation (Figure 4), and incubated at Mahi Pono within a temperature-controlled shed set to 22.8 °C for six weeks. Tar paper was inserted as a floor in each tote, and approximately 500 g of fine white sand was added before loading the totes with fruit. The sand served as a pupation medium for any flies developing in fruit. At three and six weeks, fruits were removed from each tote to sieve the sand and inspect for the presence of any fruit fly pupae. Fruit fly pupae are brown and easily detectable when present in the fine white sand. Any fruits that felt soft or were moldy were cut open to look for fruit fly larvae.

### 2.6. Statistical Analysis

For the small cage, large olfactometer cage, and sleeve cage tests, data on the number of pupae and adults emerging per kg of fruit in punctured (damaged) or undamaged limes and papaya-positive controls were analyzed using a nonparametric Wilcoxon/Kruskal–Wallis test (rank sums) with significance reported as a chi-square (χ^2^) value, and multiple comparisons were made using the Steel–Dwass method [23].

For the large-scale validation test, the level of confidence associated with collecting a specific number of fruits in the field with a predetermined acceptable level of natural infestation is given by the equation

C = 1 − (1 − *p_u_*)*^n^* (1)

where *p_u_* is the acceptable level of infestation (as a proportion of fruit) and *n* is the number of field-collected fruit [13]. Confidence levels were calculated for the number of collected limes, assuming the required efficacy for host plant resistance ([1 − *p_u_*] × 100) is 99.99%.

## 3. Results 

### 3.1. Forced Infestation in Small Cages

In small age tests, no Oriental fruit flies emerged from a total of 47 undamaged intact fruit exposed to a total of 2350 gravid flies. One Mediterranean fruit fly adult emerged from a total of 48 undamaged intact fruit exposed to 2400 gravid flies. The effect of fruit status (punctured limes, intact limes, papaya controls) was highly significant for Oriental fruit fly pupae (χ^2^ = 81.5, *p* < 0.0001) and adults (χ^2^ = 78.7, *p* < 0.0001) per kg of fruit (Table 1), as well as Mediterranean fruit fly pupae (χ^2^ = 83.3, *p* < 0.0001) and adults (χ^2^ = 79.1, *p* < 0.0001) per kg of fruit (Table 2). Damaged (punctured) limes and papaya-positive controls were highly infested by Oriental fruit fly and moderately well infested by Mediterranean fruit fly.

### 3.2. Forced Infestation in Large Olfactometer Cages

In large cage tests, no Oriental fruit flies (Table 1) or Mediterranean fruit flies (Table 2) emerged from 60 or 36 undamaged (intact) lime fruits, respectively, when exposed to a total of 1500 or 800 gravid flies. Damaged (punctured) fruits were infested by both species. The effect of fruit status (punctured limes, undamaged limes, papaya positive controls) was highly significant for Oriental fruit fly pupae (χ^2^ = 34.6, *p* < 0.0001) and adults (χ^2^ = 36.5, *p* < 0.0001) per kg of fruit (Table 1), as well as Mediterranean fruit fly pupae (χ^2^ = 18.6, *p* = 0.0002) and adults (χ^2^ = 17.5, *p* < 0.0002) per kg of fruit (Table 2). Punctured limes were a good host for both fruit fly species and were not significantly different from the papaya controls. The average infestation rate for punctured fruit in the large olfactometer cages was about 20% for Oriental fruit fly and 40% for Mediterranean fruit fly of the infestation rate in the small cages.

### 3.3. Forced Infestation in Sleeve Cages on the Tree 

Forced infestation studies in the field using sleeve cages to enclose fruit with 50 gravid females showed no infestation in undamaged intact fruit. Damaged fruit produced a high infestation rate for Oriental fruit fly and a moderately high infestation rate for Mediterranean fruit fly (Table 3). The effect of fruit status (damaged limes, undamaged intact limes) was highly significant for Oriental fruit fly pupae (χ^2^ = 18.2, *p* < 0.0001) and adults (χ^2^ = 18.2, *p* < 0.0001) per kg of fruit, as well as for Mediterranean fruit fly pupae (χ^2^ = 4.3, *p* = 0.04) and adults (χ^2^ = 4.3, *p* = 0.04) per kg of fruit (Table 3). Despite having a similar internal cage volume and the same number of flies, the infestation rate in damaged limes for Oriental fruit fly in the sleeve cage was about six times higher than in the small cage, which probably reflected the more severe damage to the fruit and greater exposure of fruit flesh in the sleeve cages (gash with a knife) compared to the small cage (multiple pin punctures).

### 3.4. Field Collection and Incubation of Fruit 

In the small-scale field collection of fruit, 4599 fruits (410.5 kg) were sampled from the tree, and one fruit fly pupa was found but it did not develop to the adult stage and could not be identified. For ground fruit, 2510 (260.3 kg) were sampled, and no fruit fly pupae were recovered (Table 4). This shows that limes that fall to the ground continue to be unattractive or unsuitable hosts, as are fruits on trees.

In the large-scale fruit collections, a total of 45,980 export-quality limes (4100 kg) were sampled from five orchard blocks and held for six weeks at room temperature for any adult fruit fly emergence. The four fruit sizes (number of fruits that fit in a 13.6 kg box) that were packed were 150 (5%), 175 (60%), 200 (30%), and 230–250 (5%). No fruit flies emerged from the commercially harvested and packed fruit (Table 4). Assuming a required efficacy of host plant resistance to infestation of 99.99%, C = 1 − (1 − 0.0001)^45,980^, and our confidence level was 99.0% that the fruit infestation rate in Persian limes was less than 0.0001 (1 in 10,000 fruit). No surface pests or hitchhikers were observed in field-collected fruit at the time of packing.

**Figure 5:**
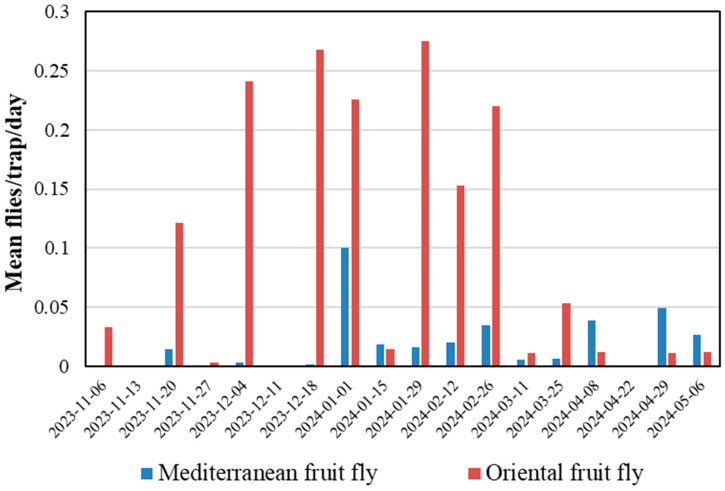
Mean fruit fly captures per trap per day in Persian limes from 64 McPhail traps in 32 orchard blocks checked biweekly at Mahi Pono.

McPhail traps captured Mediterranean fruit fly and Oriental fruit fly in the Mahi Pono orchards throughout the period of this study (Figure 5). This demonstrated that fruit flies were active in the orchard and potentially could have infested Persian limes if intact fruits were susceptible. Field collection and incubation of fruit (in small-scale and large-scale collections) was conducted from February to April 2024 (Figure 5) when both fruit fly species were being captured in traps, with mean captures of about 0.02 and 0.10 flies per trap per day for Mediterranean fruit fly and Oriental fruit fly, respectively, across all traps during that period. Another fruit fly species that is not listed as a pest of Persian lime, melon fly, was captured in the McPhail traps during this study at an average rate of 0.14 flies per trap per day but also did not infest fruit, confirming that Persian lime is not a natural host for this species.

## 4. Discussion

In our study, no Oriental fruit fly infestations were observed in export-quality undamaged green fruit in the large cage challenge tests with exposure to high numbers of fruit flies, whereas artificially damaged fruits were infested and yielded adult flies. This demonstrates that Persian limes are a potential host but conditionally a nonhost. Forced infestation studies in the field using sleeve cages to enclose fruit with a high density of fruit flies on the tree also showed no infestation in commercial-grade fruit, and no fruit flies emerged from fallen decaying fruit. Artificially damaged fruit in sleeve cage tests produced high numbers of Oriental fruit flies and relatively high numbers of Mediterranean fruit flies, demonstrating the competence of these flies to oviposit and develop in damaged fruit. A single Mediterranean fruit fly adult emerged in one small cage test, and a single fruit fly pupa emerged in one preliminary field collection test, indicating that a very low level of natural infestation may occur in the field. In these two cases, the flies may have come from lime fruits with a minor blemish or other damage that went undetected. A large-scale test with commercially harvested and packed export-quality fruit produced no Oriental fruit flies or Mediterranean fruit flies in more than 46,000 fruits. 

The International Plant Protection Convention (IPPC) has published a standard with guidelines for the determination of the host status of a particular fruit to a given fruit fly in its International Standards for Phytosanitary Treatments (ISPM) 37 entitled ‘Determination of host status of fruit to fruit flies’ [9]. In ISPM 37, a natural host is defined as a plant species or cultivar that has been scientifically found to be infested by the target fruit fly species under natural conditions and is able to sustain the fly’s development through to the emergence of viable adult flies, whereas a conditional host is one not known to occur in nature but has been found by scientific tests to support infestation by the fruit fly of interest (with development to the adult stage) under semi-natural field conditions, such as in field cages or in caged or bagged branches bearing fruit [9]. In regulations, risk managers may use the term conditional nonhost to describe regulated articles that do not pose a pest risk and to emphasize the absence of natural infestation [24]. Conditional host and conditional nonhost are synonymous. Commercial export-grade Persian lime fruit meets the definition of a conditional host in ISPM 37 for Oriental fruit fly and Mediterranean fruit fly, with negligible risk of moving fruit flies during export.

Resistance of citrus to tephritid fruit flies can be attributed to phenology (fruit maturity level) and varietal differences of the fruit [25,26], physical characteristics of the peel [27] (hardness, thickness), and chemistry of the peel, including the toxicity of essential oils to fruit fly eggs and larvae [26,28,29]. Host plant resistance can affect one life stage or multiple life stages. The specific mechanism of resistance in Persian lime has not been elucidated. Persian lime is a relatively thin-skinned type of citrus and is softly textured and, therefore, the thickness and hardness of the peel are not physical barriers to fruit fly oviposition. In the no-choice cage tests, Oriental fruit fly and Mediterranean fruit fly were mostly not interested in Persian lime fruit and spent little time examining the peel surface. A few Oriental fruit flies and Mediterranean fruit flies were observed attempting to insert their aculeus into the peel, but oviposition scars were only 2 mm deep and did not penetrate beyond the flavedo, and no eggs were deposited, suggesting that chemical characteristics of the peel (Persian limes have many oil glands in the flavedo) may have deterred oviposition [26]. In quarantine and host status studies, resistance to fruit flies can often be circumvented by puncturing the fruit skin, which has been demonstrated in poor citrus hosts such as lemons [30], oranges and tangerines [31], finger limes [4], and in the present study with Persian limes. In the no-choice cage tests, Oriental fruit fly and Mediterranean fruit fly actively examined the fruit surface and readily oviposited into the flesh of Persian limes when the peel was punctured through the flavedo and albedo to provide direct access to the pulp.

In the absence of natural infestation in commercial quality Persian limes from Hawai’i, an export protocol based on nonhost status or a systems approach using conditional nonhost status should be acceptable and have negligible risk. The key to the safe export of Persian limes using nonhost status would be effective sorting and grading procedures to ensure fruits have no damage or breaks in the skin that might allow for infestation. The standards for U.S. No. 1 and No. 2 grade Persian limes allow for 5% by count below grade fruit due to decay, stylar-end breakdown, and broken skin that is not healed [22]. Our data show that breaks in the skin make fruit more susceptible to fruit fly infestation, so this type of off-grade fruit should be eliminated at all costs to ensure the integrity of a nonhost status export protocol. Sour limes (Persian lime and Key lime, *Citrus aurantifolia* Swingle) are allowed movement into the United States from more than 40 countries without a quarantine treatment either because they are accepted as a conditional nonhost for the fruit fly species of interest or because they are grown in a fruit fly-free area [24]. The difference between these countries and Hawai’i is the presence of Oriental fruit fly and the absence of data until now on the host status of Persian limes for this species.

Despite the conditional nonhost status of Persian limes for fruit flies in Hawai’i, pest-sensitive market destinations, such as California and other citrus-growing areas, may require additional phytosanitary measures that provide extra security and a level of assurance [32]. The components of a systems approach with additional measures might include fruit maturity level (firm green fruit only) and quality (no breaks in the skin), orchard sanitation (removal of dropped fruit), fumigation to control surface pests and hitchhikers, and postharvest safeguards such as inspection for other pests, covering field bins during harvest, and pallet shrouds to prevent fruit fly access to fruit after packing. Limited sales distribution, e.g., to northern-tier states during winter months, is a more restrictive measure that is used for other fruits (e.g., avocados) exported from Hawai’i [33].

## Figures and Tables

**Table 1:** Number of Oriental fruit flies developing in Persian limes (punctured or intact) after 24 h of exposure to either 50 gravid females (small cage test) or 100 gravid females (large olfactometer cage test) and six-week incubation and holding period for adult emergence. Papaya fruits were tested similarly as a positive control.

Treatment		Total No. Fruit	Avg. Fruit Weight (g)	Total No. Pupae	Avg. (±SE) Pupae per kg Fruit	Total No. Adults	Avg. (±SE) Adults per kg Fruit	Prop. Adult Emergence
**Small cage test**								
Lime	Punctured	46	82.1	1349	358.8 ± 33.6 b	959	259.1 ± 28.9 b	0.69
	Intact	47	96.6	0	0 a	0	0 a	--
Papaya	(Control)	16	482.5	3758	515.1 ± 220.2 b	2786	377.3 ± 146.2 b	0.88
**Large olfactometer cage test**								
Lime	Punctured	48	82.1	340	85.8 ± 22.7 b	213	53.4 ± 15.5 b	0.54
	Intact	60	89.3	0	0 a	0	0 a	--
Papaya	(Control)	9	438.0	243	68.3 ± 29.1 b	229	62.1 ± 23.3 b	0.79

Means within a column followed by different letters were significantly different by the Steel–Dwass method (*p* ≤ 0.05).

**Table 2:** Number of Mediterranean fruit flies developing in Persian limes (punctured or intact) after 24 h of exposure to either 50 gravid females (small cage test) or 100 gravid females (large olfactometer cage test) and six-week incubation and holding period for adult emergence. Papaya fruits were tested similarly as a positive control.

Treatment		Total No. Fruit	Avg. Fruit Weight (g)	Total No. Pupae	Avg. (±SE) Pupae per kg Fruit	Total No. Adults	Avg. (±SE) Adults per kg Fruit	Prop. Adult Emergence
**Small cage test**								
Lime	Punctured	48	77.4	189	53.0 ± 7.0 b	124	34.5 ± 5.5 b	0.64
	Intact	34	94.0	1	0.18 a	1	0.18 ± 0.18 a	1.0
Papaya	(Control)	16	526.1	4670	597.1 ± 100.1 c	2458	330.6 ± 75.2 c	0.54
**Large olfactometer cage test**								
Lime	Punctured	36	85.0	68	23.7 ± 6.9 b	40	14.0 ± 4.2 b	0.72
	Intact	36	98.1	0	0 a	0	0 a	0
Papaya	(Control)	6	460.2	429	164.4 ± 148.3 b	338	129.7 ± 118.2 b	0.74

Means within a column followed by different letters were significantly different by the Steel–Dwass method (*p* ≤ 0.05).

**Table 3:** Number of Oriental fruit flies and Mediterranean fruit flies emerging from Persian limes (damaged or intact) after 24 h of exposure to 50 gravid females in sleeve cages over fruit on the tree in the field.

Treatment		Total No. Fruit	Avg. Fruit Weight (g)	Total No. Test Flies	Emerging from Limes			
					Total No. Pupae	Avg. (±SE) Pupae per kg Fruit	Total No. Adults	Avg. (±SE) Adults per kg Fruit
**Oriental fruit fly**								
Lime	Damaged	15	61.8	750	703	2311.7 ± 1030.7 b	550	1759.1 ± 815.6 b
	Intact	46	60.5	2300	0	0 a	0	0 a
**Mediterranean fruit fly**								
Lime	Damaged	10	82.1	500	12	39.3 ± 35.7 ab	6	19.5 ± 16.0 ab
	Intact	43	75.9	2150	0	0 a	0	0 a

Means within a column followed by different letters were significantly different by the Steel–Dwass method (*p* ≤ 0.05).

**Table 4:** Number of Oriental fruit flies and Mediterranean fruit flies emerging from export-quality green Persian limes collected in the field from the tree or the ground.

Treatment		Total No. Fruit	Total Fruit Weight (kg)	Emerging from Limes	
				No. Oriental Fruit Fly Adults	No. Mediterranean Fruit Fly Adults
**Small-scale field collection** ^1^					
Lime	Tree	4599	410	0	0 (1 pupa) ^2^
	Ground	2510	260	0	0
**Large-scale field collection** ^3^					
Lime	Tree	45,958	--	0	0

^1^ Field-collected fruit shipped to the USDA-ARS laboratory in Hilo for incubation. ^2^ One pupa was discovered that did not develop to the adult stage and could not be identified. ^3^ Field-collected fruits were packed on a packing line (washed, waxed, sorted, and sized) for simulated export and then incubated.

## Data Availability

Data can be obtained by contacting the corresponding author.
